# Supplementary material for: Surface Microbiome Profiling of Dental Elevators Using Third‐Generation Sequencing: Implications for Infection Control in Dental Practice
Source: Microbiologyopen. 2025 Dec 8;14(6):e70178. doi: 10.1002/mbo3.70178 (PMC12685768; doi:10.1002/mbo3.70178)
Supplement: Supplementary file 1 — customer_backup. [file MBO3-14-e70178-s001.zip › customer_backup/customer_backup/function_analysis/BugBase/treat/thresholds/treat.Stress_Tolerant.pdf]

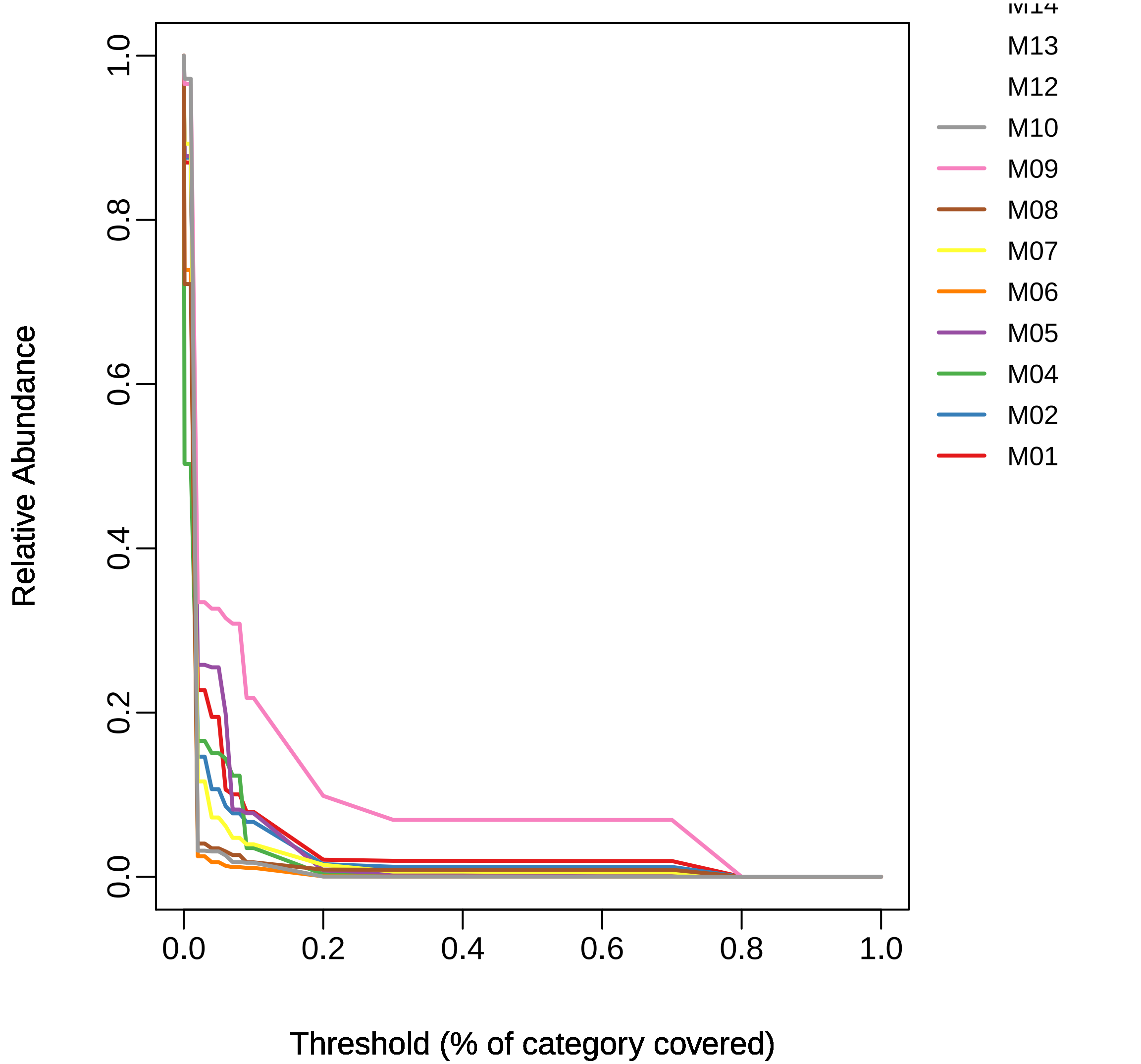

M14
M13
M12
M10
M09
M08
M07
M06
M05
M04
M02
M01
Relative Abundance
0.0
0.2
0.4
0.6
0.8
1.0
Threshold (% of category covered)
